# Bio-Mimicking, Electrical Excitability Phenomena Associated With Synthetic Macromolecular Systems: A Brief Review With Connections to the Cytoskeleton and Membraneless Organelles

**DOI:** 10.3389/fnmol.2022.830892

**Published:** 2022-03-07

**Authors:** Gary E. Wnek, Alberto C. S. Costa, Susan K. Kozawa

**Affiliations:** ^1^Department of Macromolecular Science and Engineering, Case Western Reserve University, Cleveland, OH, United States; ^2^Department of Psychiatry, Case Western Reserve University, Cleveland, OH, United States

**Keywords:** excitability, synthetic, bio-mimicking, membraneless, macromolecular

## Abstract

Electrical excitability of cells, tissues and organs is a fundamental phenomenon in biology and physiology. Signatures of excitability include transient currents resulting from a constant or varying voltage gradient across compartments. Interestingly, such signatures can be observed with non-biologically-derived, macromolecular systems. Initial key literature, dating to roughly the late 1960’s into the early 1990’s, is reviewed here. We suggest that excitability in response to electrical stimulation is a material phenomenon that is exploited by living organisms, but that is not exclusive to living systems. Furthermore, given the ubiquity of biological hydrogels, we also speculate that excitability in protocells of primordial organisms might have shared some of the same molecular mechanisms seen in non-biological macromolecular systems, and that vestigial traces of such mechanisms may still play important roles in modern organisms’ biological hydrogels. Finally, we also speculate that bio-mimicking excitability of synthetic macromolecular systems might have practical biomedical applications.

## Introduction

“An attempt to decide what constitutes the difference between living matter and dead matter cannot avoid the observation that one especial characteristic of living matter is its power to react to changes in its environment…This responsiveness is sometimes called the irritability of living matter, though the term excitability is preferable.” Thus opens Mitchell’s textbook on General Physiology (Mitchell, 1948). Changes in the environment leading to excitability are termed stimuli, and include modulation of temperature, pH, and electrical current or voltage. While stimulation and excitability are indeed manifestations of living systems, synthetic macromolecular systems can exhibit such characteristics. For example, the earliest examples of artificial muscle fibers utilized reversible pH changes of synthetic polyelectrolytes to achieve reversible motion (Katchalsky, 1949; Kuhn et al., 1950), and stimuli-responsive actuation remains a topic of great scientific and practical interest.

Less well-known are examples of synthetic macromolecular systems that under certain conditions exhibit electrical signatures reminiscent of those typically recorded from excitable cells, such as current transients in response to applied voltage gradients. Even more interesting, and also poorly disseminated in the literature, are reports of artificial macromolecular systems capable of exhibiting spontaneously generated, transient electrical signals that have time constants and amplitudes comparable to action potentials in neuronal membranes (Shashoua, 1967; Huang and Spangler, 1977). Here, we review these observations and explanations and share a few thoughts regarding potential future experiments from a polymer science perspective. We then glance at a few potential practical applications of these bio-mimetic artificial hydrogels. Finally, we speculate on the possibility that electrical excitability and signaling through biological hydrogels may have played key roles in protocells of primordial organisms, some of which might still be of functional importance in modern organisms.

## Transient Currents and Thin Membranes

**FIGURE 1:**
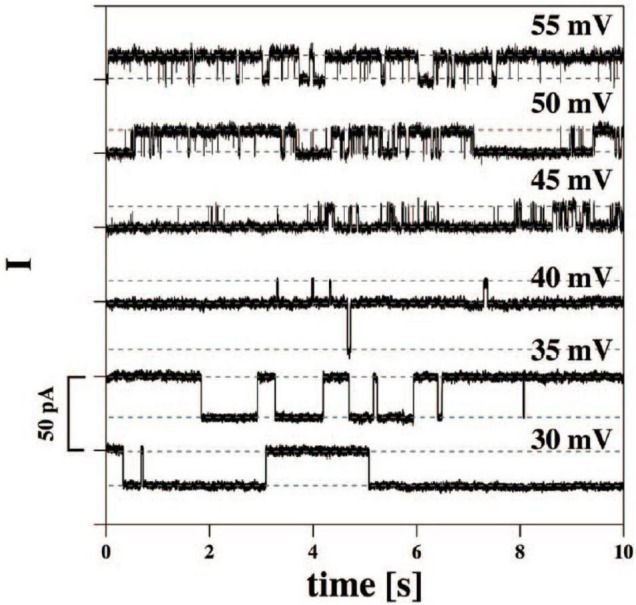
Current traces from synthetic bilayer membranes in the absence of proteins. From Blicher and Heimburg (2013).

It has been known for many decades that application of a voltage gradient across, for example, a cell membrane by voltage-clamping leads to rapid current transients through a variety of ion channels (Hille, 2001). When using the patch-clamp technique, one can record unitary opening and closing events of ionic currents in the pA scale. Less well known is that similar, square-wave current transients can also be seen with pure lipid membranes (Blicher and Heimburg, 2013; Zecchi and Heimburg, 2020). Figure 1 depicts one example of this type of current trace, which appear indistinguishable from those recorded in lipid membranes containing proteins, either from synthetic preparations or from living cells. In the case of pure lipid membranes, the existence of hydrophobic and hydrophilic pores with openings on the nanoscale has been proposed. Interestingly, the conductance of such lipid pores is of a magnitude typical of that seen in single channel proteins. Possible mechanisms for pore formation in pure lipid membranes and details of experimental conditions have been discussed in detail elsewhere (Heimburg, 2010; Blicher and Heimburg, 2013). Although the formation of pores of various sizes (subconductances) occur in a stochastic way, it has also been proposed that the application of transmembrane voltage may favor the formation of pores with a specific radius (Glaser et al., 1988; Zecchi and Heimburg, 2020).

**FIGURE 2:**
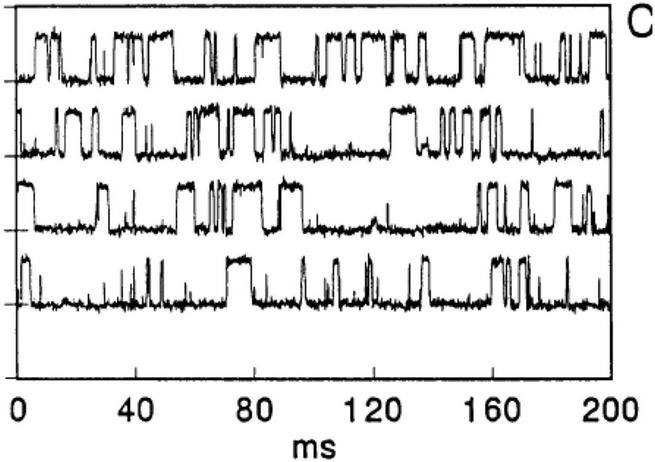
Binary gating from patch pipettes and silicone seals. From Sachs and Qin (1993).

Of particular note is that similar transients can be observed with synthetic polymers such as silicone rubber (Sachs and Qin, 1993; Figure 2), along with interesting trends in cation selectivity.

It was proposed that very thin water channels can form at the glass pipette-silicone rubber interface, providing the path for ion conduction. The high elasticity of the silicone preparation may contribute to the observations. Sachs and Qin (1993) noted that increases in voltage can lead to channel closure although no mechanistic explanation was offered. The applied voltage may have led to a decrease in thickness of the compliant silicone elastomer and, because rubbers are typically incompressible (Poisson’s ratio ≈ 0.5), thickness reduction necessarily leads to expansion in the planar dimension, as observed for silicones and other rubbery materials when used as dielectric elastomers (Madsen et al., 2016). Such lateral expansion may have served to disrupt water channels. In fact, Sachs and Qin offer a suggestion about the effect of pressure on a drop of water between two hydrophobic microscope slides: “pressing on the slides together produces a fractal array of water fingers whose shapes change when the slides are squeezed.” We interpret the change of shape of water fingers to include the possibility of capillary thread break-up under pressure and hence a significant decrease in the number of continuous channels.

It is worth noting that the interface between silicone rubber and glass is probably not filled purely with water and salts, as there is a likelihood of at least slight contamination from untethered silicone chains; this so-called sol fraction naturally results from incomplete network-forming reactions (Mazurek et al., 2019). Free silicone chains and/or cyclics have negligible water solubility but could adsorb onto the glass capillary wall. This could possibly explain the invariance of cation selectivity of the seal even when the glass was pre-treated with dimethyldichlorosilane.

It would be interesting to explore the effect of crosslink density and hence mechanical compliance on current transients as a function of applied voltage, as well as the effect of thin glass fibers embedded in a thin silicone membrane. A glass fiber/silicone composite would likely have many interfaces for the creation of numerous water channels along them. Moreover, it would be interesting to study the possibility of stabilizing water channels at the silicone-glass interface by lowering interfacial tension, and to attempt to block current spikes using molecules of variable molecular weight and hydrodynamic volume, degree of hydrophobic/hydrophilic character, and fixed charge density.

**FIGURE 3:**
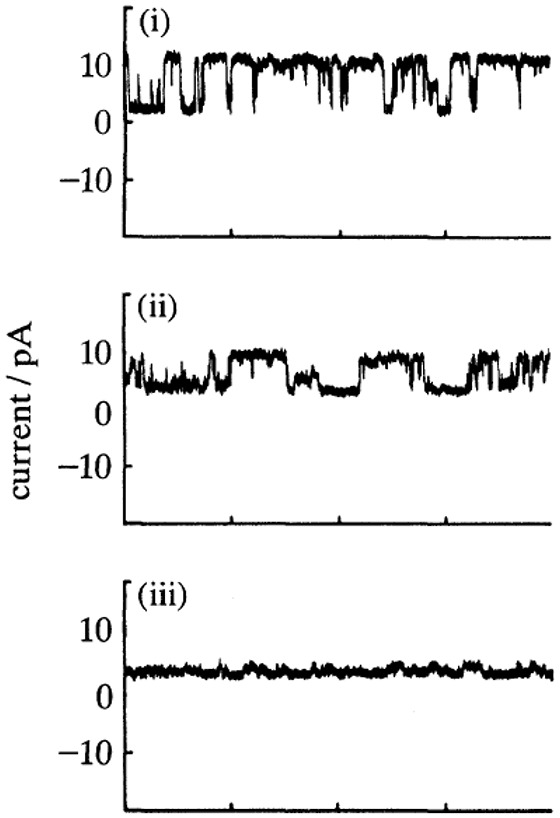
Current fluctuations across PETP nuclear filters. Filter was bathed in 0.1 M KCI, 0.005 M HEPES pH 7.4 **(i)** without or **(ii)** with 3 × 10^−4^ M or **(iii)** with 3 × 10^−3^ M. From Lev et al. (1993).

Yet another example of “gating” comes from studies of track-etched poly(ethylene terephthalate) (PET) membranes with alkali treatment, affording tethered carboxylate anions along channel walls (Lev et al., 1993; Figure 3(i)). Pore sizes were estimated to be 2–3 nm. Switching between two states of high and low conductances was observed, which was attributed to potentially anomalous conductances of aqueous solutions near solid surfaces, especially if pores as in the membranes studied have a high internal surface area-to-volume ratio. Interestingly, the current transients were sensitive to Ca^2+^, and addition of 0.3 mM Ca^2+^ modulated the response profile (Figure 3(ii)) while 3 mM Ca^2+^ (Figure 3(iii)) almost completely suppressed the transients, presumably due to abolishment of the higher-conductance state. The authors noted that addition of EDTA to chelate Ca^2+^ restored the higher-conductance state.

It is interesting to speculate about the possibility of unusual ionic conductivity in other physical configurations, specifically polyelectrolyte fibers. We note that cytoskeletal proteins such as actin are typically net anionic at physiological pH (Janmey, 1998). The track-etched, alkali-treated pores in PET may be useful as a model system to further understand ion transport along charged surfaces to complement studies on biological polyelectrolytes.

## Dynamic Properties of Polyelectrolyte Membranes: Spontaneous Spike Generation

**FIGURE 4:**
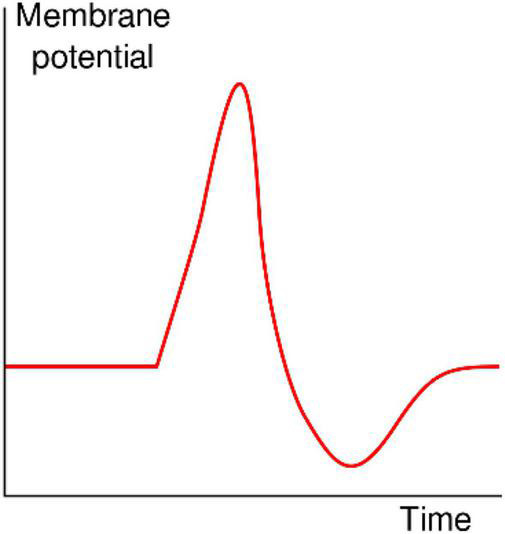
Shape of a typical action potential. The membrane potential remains near a baseline level until at some point in time, it abruptly spikes upward and then rapidly falls (https://en.wikipedia.org/wiki/Action_potential).

The classical shape of an action potential originally identified by Hodgkin and Huxley (1952) in the squid giant axon is shown in Figure 4. These stereotypic action potentials are characterized by a sharp rise in transmembrane potential until a maximum is reached, followed by a rapid drop to a potential below the initial value, which is then followed by a recovery period in which no action potentials can be fired (the refractory period) to the initial potential. It is quite interesting that the shape of the action potential and its typical time course, which are generated in axons by the sequential activation and inactivation of voltage-gated Na^+^ and K^+^ channels, can be closed recapitulated with completely synthetic systems devoid of voltage-sensitive proteins.

A pioneering example of an excitable biomimetic system is the polycation-polyanion bilayer system reported by Shashoua (1967). The polyanion was an 80/20 copolymer of acrylic acid and acrylamide (AA-ACA copolymer), adjusted to pH 4.5 (close to the pK_*a*_ of polyacrylic acid), whereas the polycation was poly(dimethylamino ethylacrylate). Protonation of the latter by the AA-ACA copolymer created a bilayer structure.

**FIGURE 5:**
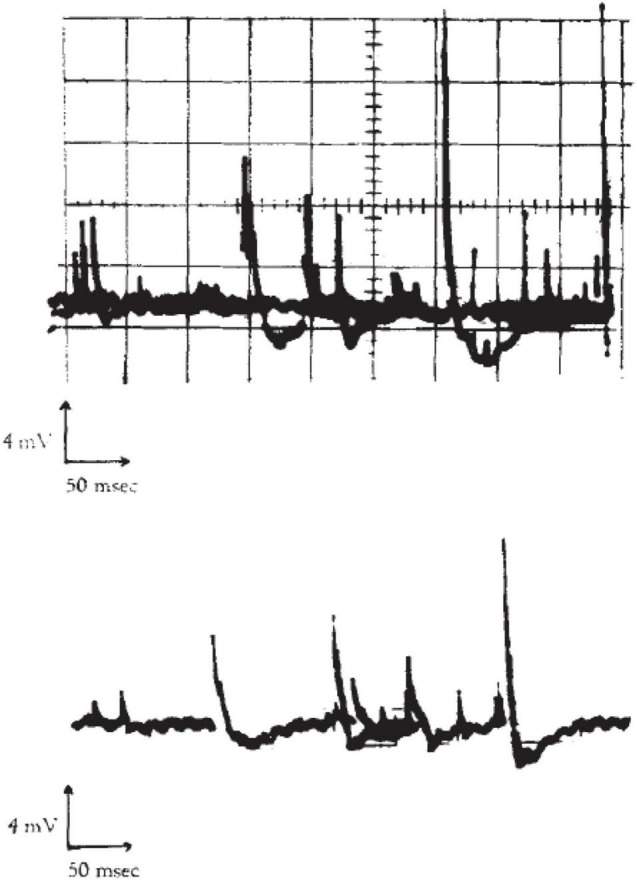
Oscilloscope traces of spikes generated by polyelectrolyte membranes. From Shashoua (1967).

**FIGURE 6:**
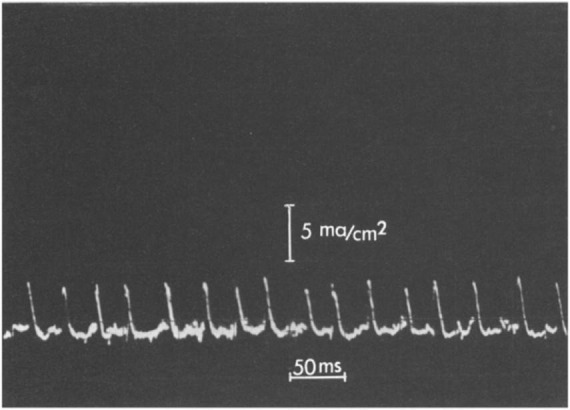
Oscillatory behavior of a poly(glutamic acid)—Ca^2+^ membrane. The amplitude scale is 5 mA/cm^2^ and the time scale is 50 ms per division. From Huang and Spangler (1977).

Application of a rising d.c. potential across the bilayer eventually led to a critical applied voltage where voltage spikes were observed (Figure 5). Remarkably, as Shashoua noted (Shashoua, 1967), the “d.c. electric field can spontaneously generate transients with time constants and amplitudes analogous to the spike potentials of neuronal membranes.” Interestingly, a polyanion-polycation bilayer membrane is not necessary for such observations. For example, Shashoua (1967) also investigated a poly(acrylic acid) membrane exposed on one side to a dilute solution of Ca(OH)_2_ and found similar transients could be observed. A variant of this system, specifically a poly(glutamic acid) membrane partially neutralized with Ca^2+^ at one face, was studied in detail (Huang and Spangler, 1977) and showed oscillations of membrane current (Figure 6). Shashoua (1974) further investigated the spiking phenomenon in a broader series of polyelectrolyte bilayers including, for example, DNA and poly(lysine hydrobromide).

**FIGURE 7:**
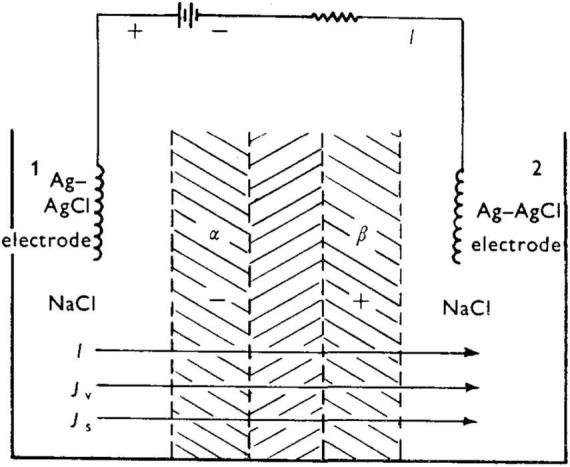
Experimental set-up of Shashoua (1967) leading to a rapid succession of spike oscillations. From Katchalsky and Spangler (1968).

A preliminary proposal to explain the results in Figure 5 was put forth by Katchalsky (1949) (noted in Shashoua (1967) paper), which was subsequently refined by Katchalsky and Spangler (1968). Figure 7 is a sketch of the experimental set-up. Before summarizing the analysis, it is appropriate to review four general aspects of the behavior of polyelectrolytes. First, a polyelectrolyte chain has fixed charges (for example, carboxylate units) balanced by counterions which are mobile. As such, membranes with fixed charges will be permselective toward the mobile counterion, and will tend to reject co-ions of similar sign. Second, polyelectrolyte networks can undergo substantial swelling in water owing to an osmotic driving force that works to dilute the mobile counterions (e.g., Na^+^ in the case of an anionic polyelectrolyte).

Swelling leads to stretching of polyelectrolyte chains, and swelling stops when the osmotic pressure is balanced with the increased elastic strain energy of the constituent chains. While the polyanion-polycation bilayer described by Shashoua (1974) is not covalently crosslinked, it is an insoluble material owing to the neutralization of the polycation by the polyanion at an interphase between the two, and thus behaves in thin film form as a network. Third, the degree of swelling will decrease in the presence of a bathing aqueous salt as the result of a decrease of osmotic pressure between the gel interior and the surrounding fluid, with the polyelectrolyte chains reverting to a more coiled vs. extended conformations. Fourth, increasing salt tends to lower permselectivity due to electrostatic screening of fixed charges.

**FIGURE 8:**
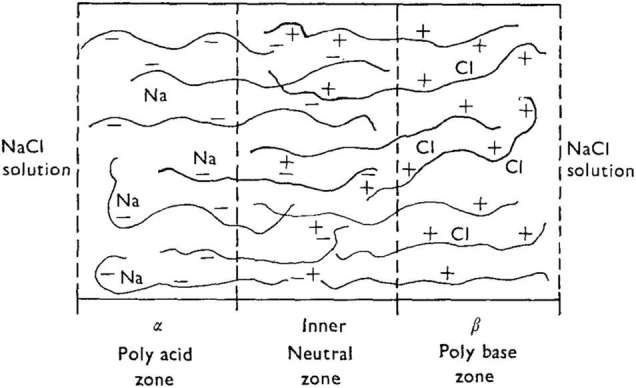
Schematic of a bilayer structure of Shashoua (1967) with an anionic polyelectrolyte (polyacid) and a cationic polyelectrolyte (polybase) and an interfacial region (inner neutral zone) where the two polyelectrolytes slightly interdiffuse. From Katchalsky and Spangler (1968).

Taking Shashoua’s (1967) polyanion-polycation bilayer as an example, and prior to application of an external voltage (i.e., at “rest”), the bilayer will be swollen to some equilibrium degree in the presence of aqueous NaCl. There exists a thin “inner neutral zone” resulting from compensation of opposite fixed charges, which, as stated earlier, also serves to confer physical integrity to the bilayer. Figure 8 shows the initial events upon imposition of a voltage (negative polarity at the polyanion side) will lead to transport of mobile ions, Na^+^ and Cl^–^). The direction of the current, I, is shown. (For a theoretical description of the fluxes J_*v*_ and J_*s*_, the reader is referred to the Appendix in Katchalsky and Spangler, 1968). Because a polyelectrolyte with a fixed charge is permselective for counterion of opposite charge, the resulting current serves to transport Na^+^ through the polyacid zone into the neutral zone, and Cl^–^ from the polybase zone to the latter.

Three interdependent events are proposed to occur with the following consequences:

(1) the build-up of NaCl in the neutral zone further increases the osmotic swelling, thereby dilating the neutral zone and increasing the hydrostatic pressure; (2) the increased salt concentration is expected to promote polyelectrolyte chain contraction and a thinning of the membrane; and (3) the salt permeability of the thinned membrane is expected to increase, principally due to a decrease in permselectivity resulting from screening of fixed charges. In other words, the polyelectrolytes will become more permeable to co-ions (Cl^–^ in the case of the polyacid, and thus more permeable to NaCl overall rather than to predominately Na^+^).

The trigger point for the spike and regeneration to continue spiking can be described as follows. Salt concentration (NaCl in this case) will increase within the membrane as the result of fluxes of both ions driven by the current. This will lead to water diffusion into the interior of the membrane, driven by the osmotic gradient. The increase in salt concentration will also lead to contraction of polyelectrolyte chains, increasing the hydrostatic pressure within the membrane. According to Katchalsky and Spangler (1968), if the hydrostatic pressure resulting from de-swelling of the polyelectrolytes becomes greater than the osmotic driving force, water will be forced out of the membrane in what is in principle reverse osmosis. This would serve to further increase the salt concentration in the membrane. However, since the polyelectrolytes progressively lose permselectivity as salt concentration increases, salt will begin to leave the membrane due to the concentration gradient, and the system effectively re-sets, enabling the process to repeat provided a potential is constantly applied.

Katchalsky and Spangler (1968) estimated the critical current, about 15 mA/cm^2^, at which oscillations should appear which was in the range of experimental measurements of Shashoua (1974). They also estimated a frequency of oscillation of about 125 Hz, and an oscillatory period of about 8 ms, which are of the correct order of magnitude.

## The “Channel-Less World”

Although ion channels are prevalent in modern organisms of all three domains (Archaea, Bacteria, and Eukarya) (Szabo et al., 1993; Mackrill, 2012; Jiang et al., 2020), it is possible that ancestral organisms, lacking a typical cell membrane, may have been solely dependent of channel-less mechanisms akin to those observed in synthetic macromolecular systems to express excitability in response to environmental stimuli. This primitive form of excitability would eventually become the template for the emergence and evolution of pore-containing transmembrane proteins. In this hypothetical scenario, the ability of specialized ion channels to sense physical (e.g., voltage, pressure, temperature) and chemical (e.g., neurotransmitters, pH, divalent cations) would have allowed for superior selectivity, control, spatial and temporal segregation, and modulation of excitability. Such exquisite level of control would eventually become essential for the fitness and survival of modern unicellular and multicellular organisms, which explains the vast number of genetic and acquired (autoimmune and inflammatory) channelopathies affecting human health (Capecchi et al., 2019; Allen et al., 2020; Kessi et al., 2021; Maggi et al., 2021; Mantegazza et al., 2021).

## “Vestigial Channel-Less Excitability” in Biological Hydrogels Present in Modern Organisms

In recent published findings (Cantero et al., 2018) indicated that electrical oscillations are an intrinsic property of neuronal microtubule bundles. These authors describe these structures as “bio-electrochemical transistors that form non-linear electrical transmission lines.” They also hypothesize that electrical oscillations carried by microtubule bundles “may have important implications in the control of various neuronal functions, including the gating and regulation of cytoskeleton-regulated excitable ion channels and electrical activity that may aid and extend to higher brain functions such as memory and consciousness.” This view parallels and extends previously published evidence that another important component of the cytoskeleton, actin filaments, can also act as electrical transmission lines for ionic currents along their lengths, which may be the basis of a wide variety of physiological effects (Lin and Cantiello, 1993; Tuszynski et al., 2004; Priel et al., 2006; Hunley and Marucho, 2021). This is because actin filaments, considered as highly charged, one-dimensional polyelectrolytes, can have significant ionic movements along the fiber axis connected to “counter-anionic clouds,” affording propagation of soliton-like, traveling ionic waves.

Over the past decade, a growing number of distinct intracellular membraneless organelles have become the focus of intense study by biophysicists, cell and molecular biologists, as well as translational researchers (Feng et al., 2019; Ryan and Fawzi, 2019). These specialized structures are formed mostly from proteins and RNAs, through liquid–liquid phase separation. Similar to microtubules and actin filaments, these supramolecular structures share many physical and chemical properties with synthetic polyanionic hydrogels.

The current list of membraneless organelles include, but is not limited to, germ granules, stress granules, nucleoli, centrosomes, and postsynaptic densities in neurons (Feng et al., 2019). In these compartments, due to the lack of physical separation, molecules can diffuse in and out the surrounding bulk solution more freely than in membrane-enclosed organelles. Still, sharp concentration gradients can be maintained between this biological hydrogel interior and the liquid, diluted exterior. These reaction machineries can reversibly assemble and disassemble within as little as a few seconds, and act as the effector elements controlling various specific biological functions.

Although not typically included in the list of membraneless organelles, condensed chromatin, which is formed by the association of nuclear DNA with histones, can self-associate into nucleosome arrays, and both euchromatin and heterochromatin have shown solid-like (compact hydrogels) behavior under physiological conditions (Strickfaden et al., 2020). This solid chromatin scaffold supports liquid-liquid phase separation of chromatin binding proteins. Interestingly, histone acetylation disperses the structure, but does not liquefy chromatin.

All the supramolecular structures described in the previous paragraphs retain many of the properties hypothesized to exist in ancestral organisms (Matveev, 2019; Early phase-based rather than membrane-based notions of cell organization of G. N. Ling, championed by G. H. Pollack and others, are discussed.). Similar to microtubules and actin filaments, it is possible that many of these structures continue to display a type of “vestigial” form of excitability that could have been the dominant mode of rapid electrical signaling in a primordial, “channel-less world.” The coordination of ionic current through circuits comprised of these structures could potentially allow for faster physiological responses than would ever be possible through chemical signaling, with tubular structures serving as the conduits of information (in the form of changes of local ionic concentration) and membraneless organelles as the effectors or hubs in these putative intracellular macromolecular electrical circuits.

Whether membraneless organelles are excitable in the strictest sense is a question that has not yet been answered. However, many of these structures are capable of phase transitions and can dilate or shrink in size depending on the ionic composition of their immediate microenvironment. Small and compact structures are less likely to allow for the free movement of macromolecules (RNA and enzymes) than larger, disordered and more porous structures. As noted by Ryan and Fawzi (2019), “the proposed biophysical functions of (membraneless organelles) seem to fall into a few broad categories […], including sequestering molecules (e.g., preventing RNA translation, as in transport and stress granules), regulating reaction kinetics by concentrating components, and altering reaction specificity by sequestering specific components to increase their local concentration (as in P-bodies) […].” Therefore, it is easy to argue that many (if not most) membraneless organelles are excitable in the sense that they are able to respond to the activity and distribution of electrical charges around them. Moreover, given that RNA and most enzymes contained in membraneless organelles tend to possess a net negative charge at intracellular pH, the sequestering or release of these molecules into the cytosol would also represent a charge transport phenomenon (i.e., an electrical current).

There is growing interest in coacervate-based, membraneless organelle and protocell models (Donau et al., 2020), and we note that the polyanion-polycation bilayer membrane discussed earlier (Shashoua, 1967) exhibiting excitability is effectively a bilayer complex coacervate. It would be interesting to explore excitability phenomena in these systems where there is considerable opportunity to selectively tune macromolecular structure, composition, and fixed charge density.

## Potential Applications

There are many practical applications that could potentially emerge from a deeper knowledge of the electrical excitability phenomena associated with synthetic macromolecular systems. The most straightforward of them would involve the fabrication of minimal analogs of neuroexcitation and neuroconduction using synthetic macromolecular systems, which could be put to use in areas of regenerative medicine, such as in nerve repair. It is also possible to conceive of synthetic biological entities that would incorporate synthetic hydrogels as part of their signaling/excitability machinery. Finally, the fields of neurodegeneration and aging have studied for a long time the role of amyloid and microtubules in the pathogenesis of various neurological disorders, such as Alzheimer’s disease (Brandt and Bakota, 2017). More recently, these fields have started to appreciate the role of membraneless organelles in various forms of neurodegeneration, such as amyotrophic lateral sclerosis and frontotemporal dementia (Babinchak and Surewicz, 2020). In addition, recently the idea that adenosine triphosphate (ATP) may act to keep proteins soluble in the cytosol (acting as a hydrotrope) has been proposed (Patel et al., 2017). Millimolar concentrations of ATP would prevent the protein-rich cytoplasm from becoming a gel and would also control the formation of membraneless organelles. Based on this scenario, Patel et al. (2017) speculated that “age or mitochondrial impairment, […] could lead to increased aggregation and consequently neurodegenerative decline […].” Given that this ATP property as a hydrotrope can be modeled in synthetic gels such as those formed by poly(acrylic acid) (Tasaki, 2002, 2005; Kozawa et al., 2021) one could potentially use such synthetic systems to reproduce in the lab many of the conditions leading to the onset of gelation of these macromolecular systems and test different physical and chemical strategies to prevent it from happening using high throughput assays. We also foresee the use of synthetic polyanionic gels as cytosol mimics in artificial cells for applications in the rapidly growing field of synthetic biology (Buddingh and van Hest, 2017).

## Conclusion

Until recently, the study of electrical excitability phenomena associated with synthetic macromolecular systems has been mostly relegated to footnotes of biophysical and physiological texts. However, there is an emerging appreciation of the fact that these systems share many similarities with biological macromolecular systems, including, but not limited to, self-association, formation of static and dynamic ionic gradients (which resemble those seen across biological membranes), and the powerful effects of divalent cations such as Ca^2+^ (Mussel et al., 2019). The study of such properties has the potential to open brand new avenues of exploration through the use of these synthetic systems to model biological processes that are difficult or impossible to access using currently available techniques. In addition, a deeper understanding of the mechanisms underlying excitability in synthetic macromolecular systems may open new and powerful applications to fields as diverse as synthetic and evolutionary biology, neurodegeneration, aging, and regenerative medicine.

## Author Contributions

GW and SK led writing in introduction and sections on transient currents and dynamics of polyelectrolyte membranes. AC led writing on the channel-less world, vestigial channel-less excitability, and potential applications. All authors contributed to editing and refining the final manuscript.

## Conflict of Interest

The authors declare that the research was conducted in the absence of any commercial or financial relationships that could be construed as a potential conflict of interest.

## Publisher’s Note

All claims expressed in this article are solely those of the authors and do not necessarily represent those of their affiliated organizations, or those of the publisher, the editors and the reviewers. Any product that may be evaluated in this article, or claim that may be made by its manufacturer, is not guaranteed or endorsed by the publisher.
